# Guillain-Barré Syndrome Following Chickenpox With Multiple Cranial Nerve Palsies and Cerebrospinal Fluid Pleocytosis

**DOI:** 10.7759/cureus.15388

**Published:** 2021-06-02

**Authors:** Kandan Balamurugesan, Chandni Chandramouli, Abdoul Hamide

**Affiliations:** 1 Medicine, Jawaharlal Institute of Postgraduate Medical Education and Research, Puducherry, IND

**Keywords:** guillain-barré syndrome, varicella, chickenpox, csf pleocytosis, cranial nerve palsy

## Abstract

Guillain-Barré syndrome (GBS) is a rare complication of chickenpox. All cases of post-varicella GBS published in the literature have been associated with the classical albuminocytological dissociation. We report the case of a 48-year-old male with flaccid areflexic quadriparesis and bilateral seventh, ninth, tenth, and twelfth cranial nerve palsies 10 days after chickenpox. Cerebrospinal fluid (CSF) analysis done in the second week showed marked lymphocytic pleocytosis. Electroneurographic studies were suggestive of acute inflammatory demyelinating polyradiculopathy. He had near-total neurological recovery with intravenous immunoglobulin. Our case demonstrates that GBS can occur after primary varicella infection, and marked CSF pleocytosis can be an additional feature.

## Introduction

Neurological complications of varicella-zoster virus (VZV) infection include meningoencephalitis, cerebellitis, myelopathy, postherpetic neuralgia, vasculopathy, acute demyelinating encephalomyelitis, and, rarely, Guillain-Barré syndrome (GBS) [1]. GBS following varicella infection is reported more commonly after secondary reactivation (herpes zoster) rather than primary varicella infection (chickenpox). Multiple cranial nerve palsies, frequently a consequence of vasculopathy, have also been reported [2]. Almost all cases of varicella-related GBS published in the literature have been associated with the classical albuminocytological dissociation. We describe a case of postprimary varicella GBS with multiple cranial nerve palsies with marked cerebrospinal fluid (CSF) pleocytosis in a 48-year-old male who recovered completely after treatment with intravenous immunoglobulin.

## Case presentation

A 48-year-old male presented to the Emergency Department (Jawaharlal Institute of Postgraduate Medical Education and Research, Puducherry) with difficulty swallowing 10 days after the onset of a chickenpox rash. He also had difficulty speaking, dribbling of food from both sides of the mouth, loss of taste, and inability to close his eyes on the left side more than the right side. Five days later, he developed numbness over both lower limbs and a mild decrease in sensation over the lower limbs, followed by ascending quadriparesis without bowel or bladder involvement and no band-like sensation, back pain, or neck pain. He had no breathing difficulty or neck muscle weakness. There was no history of altered sensorium or seizures. He had no antecedent history of respiratory or diarrheal illness, animal bites, or vaccination. He had no prior comorbidities, and there was no significant family history.

**Figure 1:**
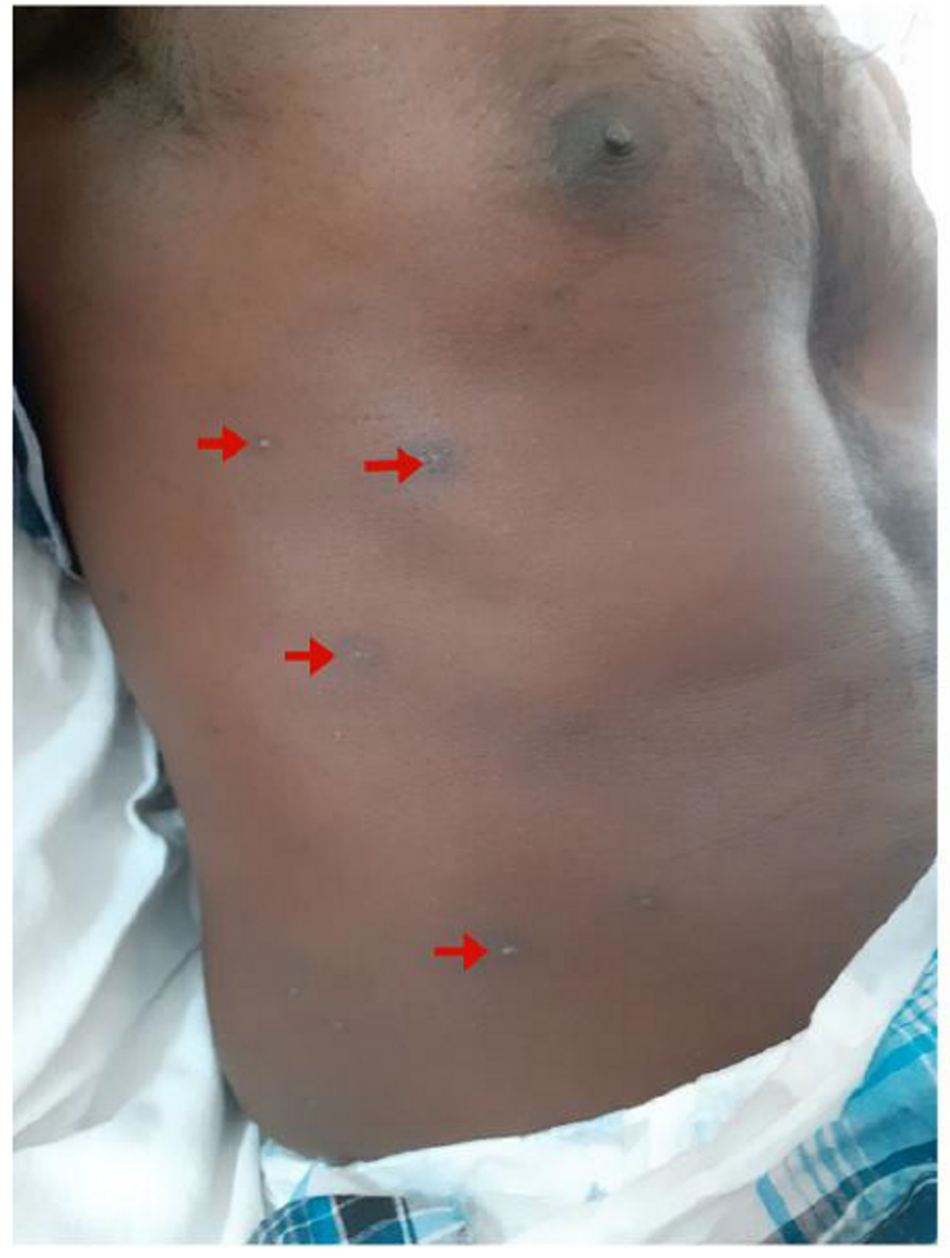
Chickenpox rash over the trunk with arrows showing where scabs have separated.

**Figure 2:**
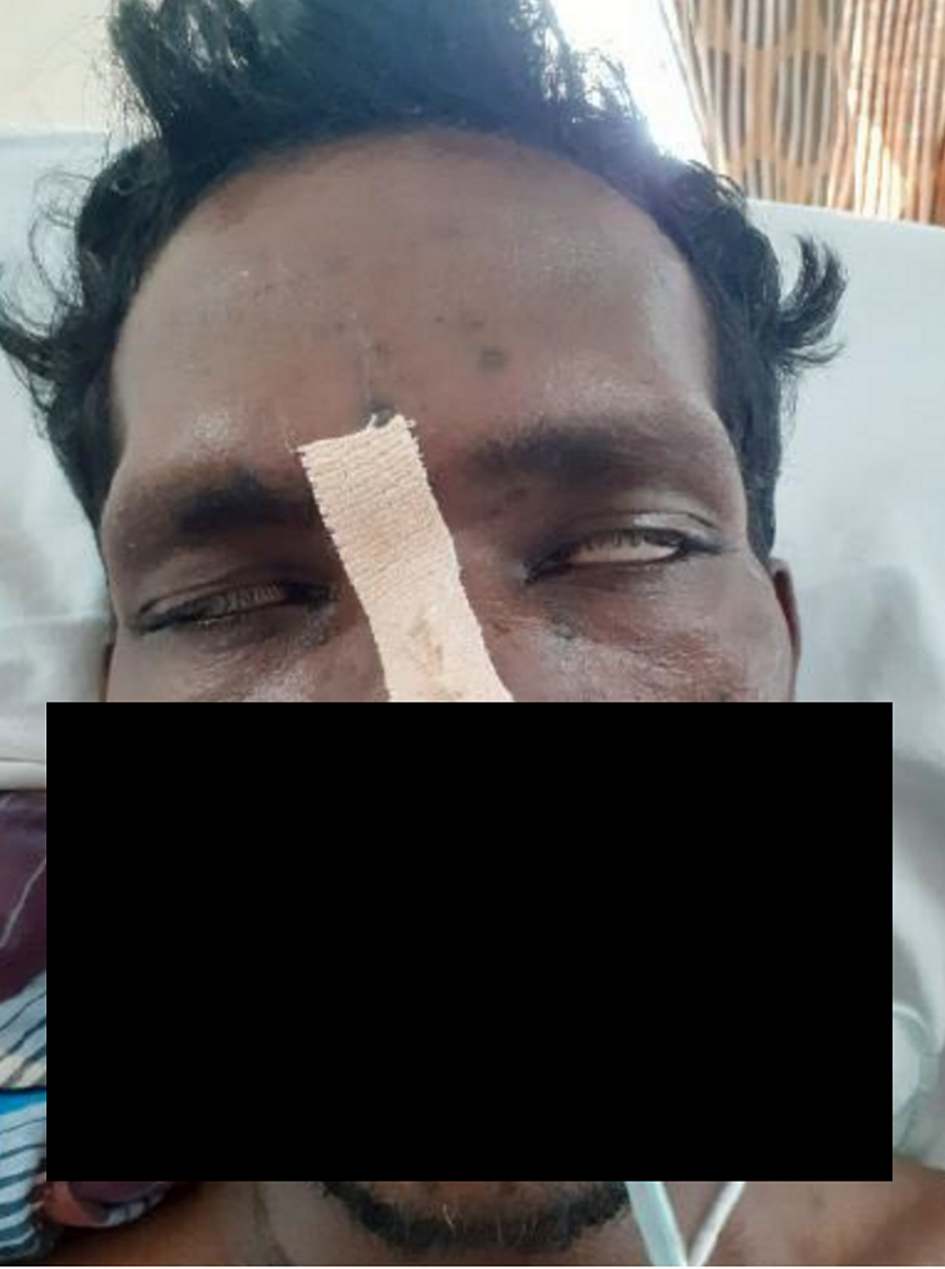
Bilateral incomplete eye closure and Bell’s phenomenon suggestive of lower motor neuron facial palsy.

His general examination was unremarkable, except for healed chickenpox lesions (Figure 1). In contrast, his neurologic examination revealed a bilateral lower motor neuron type of facial nerve involvement (Figure 2) with bilateral ninth, tenth, and twelfth cranial nerve palsies. He had flaccid areflexic quadriparesis with predominant proximal muscle involvement (power in upper limbs 3/5 proximally and 4/5 distally, lower limb 2/5 proximally and 3/5 distally). There was a distal sensory loss for fine touch, vibration, and joint position sense in all four limbs with preservation of pain and temperature. Examination of the fundus did not reveal any evidence of papilloedema.

**Figure 3:**
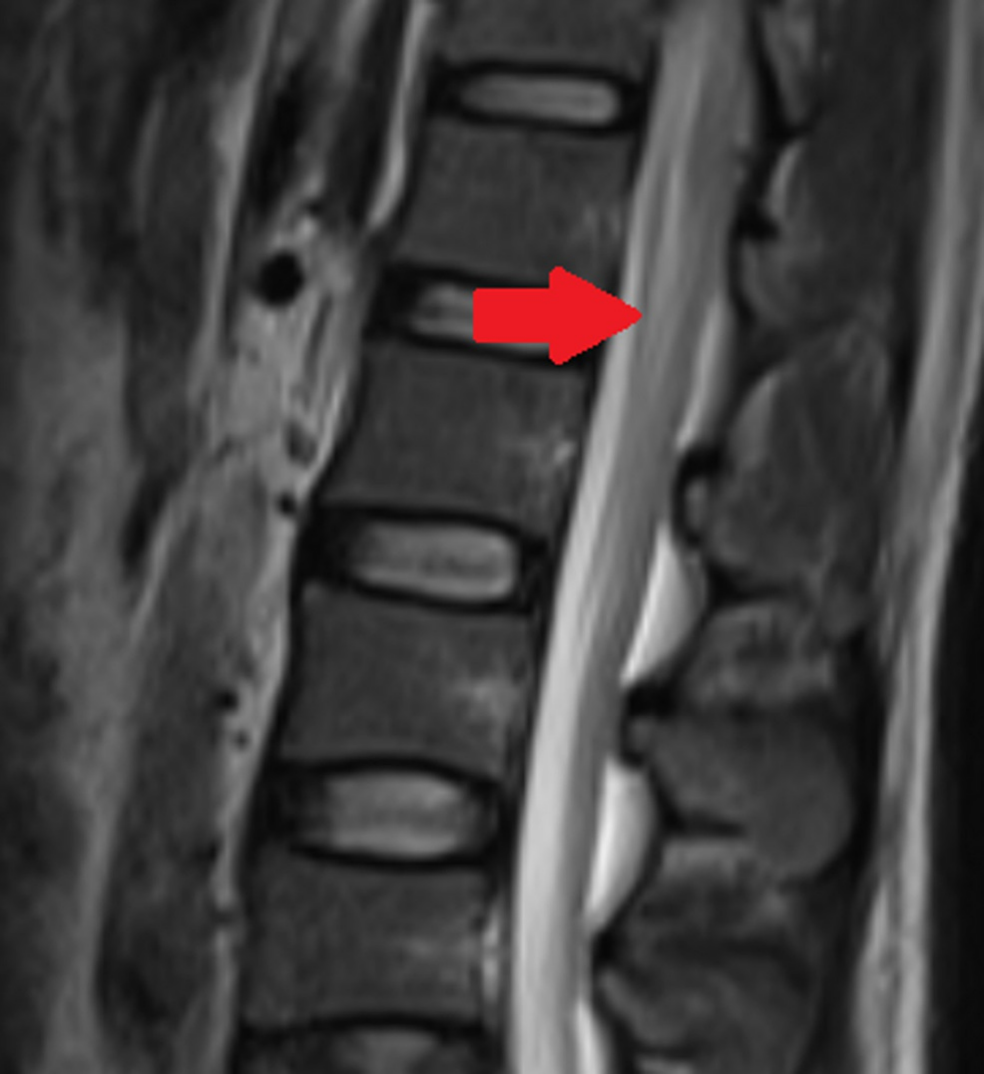
MRI spine showing surface enhancement over cauda equina nerve roots.

**Figure 4:**
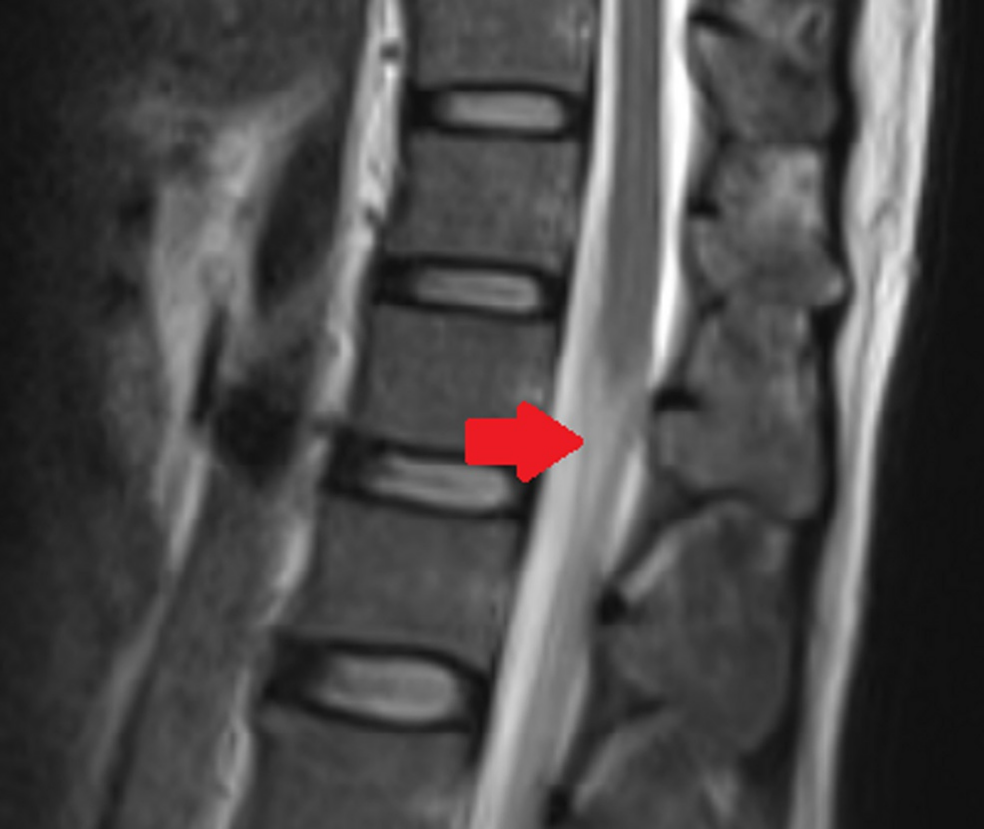
MRI spine showing surface enhancement over the conus medullaris.

CSF analysis revealed lymphocytic pleocytosis of 620 cells/mm^3^; protein was 220 mg/dL, and glucose was 81 mg/dL, with a corresponding blood glucose of 110 mg/dL. CSF polymerase chain reaction (PCR) for VZV was negative. Other biochemical and hematological investigations were within normal limits. A nerve conduction study showed decreased conduction velocity, prolonged distal latency, and mildly reduced amplitudes in the bilateral median, ulnar, tibial, and peroneal nerves, as well as facial nerves, suggesting acute inflammatory demyelinating polyneuropathy. Facial nerve study showed bilateral reduced compound muscle action potential amplitude and prolonged latency. He was diagnosed to have acute inflammatory demyelinating polyneuropathy based on the clinical and electroneurographic findings. Although the clinical and electroneurographic picture was consistent with GBS, along with a temporal correlation with antecedent varicella infection, the presence of marked CSF pleocytosis to the tune of 620 cells/mm^3^ was not typical of GBS. HIV serology was negative. His serum angiotensin-converting enzyme level was normal. An MRI of the spine showed surface enhancement over the cauda equina roots (Figure 3) and the conus medullaris (Figure 4), suggestive of demyelinating polyradiculopathy. There were no features to suggest neurosarcoidosis or tuberculosis. Therefore, the possibility of varicella-related radiculomyelitis was considered. However, there were no features of meningitis or myelitis on the MRI to explain the CSF pleocytosis. The rest of the brain and spine were normal on imaging. His CSF culture was sterile and negative for the Venereal Disease Research Laboratory test, GeneXpert for *Mycobacterium tuberculosis* test, and malignant cytology. As the patient presented during the coronavirus disease 2019 (COVID-19) pandemic, a throat swab PCR for COVID-19 was performed and found to be negative. Antiganglioside antibody was not done in our patient.

He was treated with intravenous immunoglobulin (2 g/kg body weight) over five days. Repeat CSF analysis on day three after initiating treatment showed 60 cells/mm^3^ (all lymphocytes); protein was 139 mg/dL, and glucose was 63 mg/dL (blood sugar: 100 mg/dL). At follow-up after six weeks, his power in all four limbs was 5/5, and his cranial nerve palsies had improved completely. As the patient had a complete neurologic recovery, we did not do a repeat nerve conduction study.

## Discussion

Neurological complications of VZV infection include meningoencephalitis, cerebellitis, myelopathy, postherpetic neuralgia, and, rarely, GBS [1]. GBS following VZV infection is reported more commonly after herpes zoster than chickenpox. Multiple cranial nerve palsy, frequently a consequence of vasculopathy, has also been reported [2].

A hallmark of GBS is albuminocytological dissociation: the presence of elevated protein in an otherwise acellular CSF. Our patient presented with clinical and electroneurographic features consistent with GBS without albuminocytological dissociation. This dissociation differentiates GBS from infectious and inflammatory causes of flaccid paralysis and is regarded as a core diagnostic criterion. Increased CSF cellularity in GBS should ordinarily prompt consideration of conditions like HIV, sarcoidosis, and leukemia. CSF pleocytosis can be observed in VZV infection-associated vasculopathy, myelitis, and meningoradiculitis, which were ruled out in our patient by MRI [1].

Only a few cases of CSF pleocytosis in GBS have been reported. Doctor et al. described pleocytosis (72 cells/mm^3^) in a patient with *Campylobacter jejuni*-related GBS and anti-GM1 antibodies. They identified only five other cases of pleocytosis in GBS in the literature [3]. In an independent case series of post-VZV neurological complications, Tatarelli et al., Paul et al., and Vangiliappan et al. reported cases of GBS with albuminocytological dissociation [4-6]. To our knowledge, this is the first case of primary VZV-related GBS with marked pleocytosis in the literature. This case is also notable for bilateral facial and lower cranial nerve involvement as a consequence of CNS vasculopathy.

## Conclusions

GBS is an acquired inflammatory demyelinating polyneuropathy that usually occurs following infections. In most cases, GBS is characterized by albuminocytological dissociation and bilateral lower motor neuron type of seventh cranial nerve palsy. GBS associated with CSF pleocytosis and multiple cranial nerve palsies, viz seventh, ninth, tenth, and twelfth, following primary varicella infection is quite rare, and in the literature review, case reports like this are scant. Hence, we report GBS following primary varicella infection associated with marked CSF pleocytosis and multiple lower cranial nerve palsies as well as bilateral lower motor neuron type of facial nerve palsy rarity and unique presentation. The take-home message would be that GBS can occur even with marked CSF pleocytosis and multiple cranial palsies following an infection like chickenpox in this patient.
